# Draft genome sequence of *Paenibacillus sp.* strain A2

**DOI:** 10.1186/s40793-015-0125-7

**Published:** 2016-01-26

**Authors:** Beiwen Zheng, Fan Zhang, Hao Dong, Lujun Chai, Fuchang Shu, Shaojin Yi, Zhengliang Wang, Qingfeng Cui, Hanping Dong, Zhongzhi Zhang, Dujie Hou, Jinshui Yang, Yuehui She

**Affiliations:** State Key Laboratory for Diagnosis and Treatment of Infectious Diseases, Collaborative Innovation Center for Diagnosis and Treatment of Infectious Diseases, The First Affiliated Hospital, College of Medicine, Zhejiang University, Hangzhou, China; Key Laboratory of Marine Reservoir Evolution and Hydrocarbon Accumulation Mechanism, School of Energy Resources, China University of Geosciences, Beijing, China; College of Chemistry and Environmental Engineering, Yangtze University, Jingzhou, China; Institute of Porous Flow & Fluid Mechanics, Chinese Academy of Sciences, Langfang, China; State Key Laboratory of Heavy Oil Processing, China University of Petroleum, Beijing, China; College of Life Sciences, China Agricultural University, Beijing, China

**Keywords:** *Paenibacillus sp.* strain A2, Genome, Hiseq2000, Sulfonate biodegradation

## Abstract

*Paenibacillus sp.* strain A2 is a Gram-negative rod-shaped bacterium isolated from a mixture of formation water and petroleum in Daqing oilfield, China. This facultative aerobic bacterium was found to have a broad capacity for metabolizing hydrocarbon and organosulfur compounds, which are the main reasons for the interest in sequencing its genome. Here we describe the features of *Paenibacillus sp.* strain A2, together with the genome sequence and its annotation. The 7,650,246 bp long genome (1 chromosome but no plasmid) exhibits a G+C content of 54.2 % and contains 7575 protein-coding and 49 RNA genes, including 3 rRNA genes. One putative alkane monooxygenase, one putative alkanesulfonate monooxygenase, one putative alkanesulfonate transporter and four putative sulfate transporters were found in the draft genome.

## Introduction

*Paenibacillus* is a genus of aerobic, Gram-positive, rod-shaped, and endospore forming bacteria, formerly included within the genus *Bacillus**,* but was proposed as a separate genus in 1993 on the basis of its unique distrinctive phenotypic and genotypic features [1]. Strains in this genus have been detected in a variety of environments including soil, water, rhizosphere, vegetable matter, forage and insect larvae, as well as clinical samples [2–6]. One hundred and forty nine species and four subspecies have previously been recorded in the genus *Paenibacillus*. These bacteria produce various metabolites, which can catalyze a wide variety of synthetic reactions in fields ranging from cosmetics to biofuel production and have gained importance in agriculture, industrial and medical applications [7].

Surfactant flooding is an important form of EOR to reduce the interfacial tension between oil and water to an ultra-low value [8]. Until now, sulfonate surfactants have been widely adopted as flooding agents in EOR in some oilfields under different geological conditions [9]. Surfactant flooding technology has been widely applied in the Daqing oilfield (China), and in our previous work three indigenous bacteria were isolated as crude-oil degrading species that enhance oil recovery [10]. While screening hydrocarbon-degrading bacteria previously, we isolated a *Paenibacillus sp.* strain A2 from a mixture of formation water and petroleum in Daqing oilfield. Strain A2 grows aerobically with tetradecane and hexadecane as the sole carbon and energy source, and was also found to have a capacity to metabolize organosulfur compounds. To date, data on the genetic basis of metabolizing hydrocarbon and sulfur compounds in genus *Paenibacillus* are only sparsely available. To gain insight into the nature and genomic plasticity of this strain from a unique niche its genome was sequenced and here we report a summary classification and genome annotations for *Paenibacillus sp.* strain A2.

## Organism information

### Classification and features

**Fig. 1:**
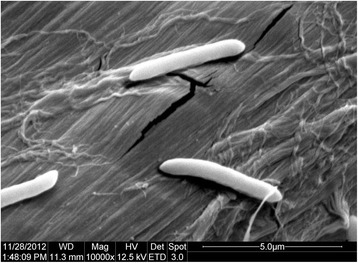
Scanning electron micrograph of cells of strain A2. Bar: 5.0 μm

*Paenibacillus sp.* strain A2 was isolated from a mixture of formation water and petroleum in Daqing oilfield, China, in March 2012. It is a Gram-positive bacterium that can grow on LB broth agar at 37 °C. Cells of strain A2 are rod-shaped, showed a diameter ranging 0.4–0.7 μm and from 1.5 to 3.6 μm long, occurring predominantly singly (Fig. 1). Growth occurs under aerobic condition. The optimum temperature for growth is 37 °C, with a temperature range of 15–45 °C (Table 1). Cell morphology, motility and sporulation were examined by using scanning electron microscopy (Quanta 200, FEI Co., USA).

**Table 1:** Classification and general features of *Paenibacillus sp.* strain A2

MIGS ID	Property	Term	Evidence code^a^
	Classification	Domain: *Bacteria*	TAS [31]
		Phylum: *Firmicutes*	TAS [32–34]
		Class: *Bacilli*	TAS [35, 36]
		Order: *Bacillales*	TAS [37, 38]
		Family: *Paenibacillaceae*	TAS [36]
		Genus: *Paenibacillus*	TAS [1, 39–42]
		Species: *Paenibacillus* sp.	IDA
		Strain: A2	IDA
	Gram stain	Positive	IDA
	Cell shape	Rod-shaped	IDA
	Motility	Motile	IDA
	Sporulation	Spore-forming	IDA
	Temperature range	Mesophile	IDA
	Optimum temperature	37rb	IDA
	pH range; Optimum	5.0–9.0; 6.0–8.0	IDA
	Carbon source	Glucose, xylose, mannitol, arabinose	IDA
	Energy source	Glucose, xylose, mannitol, arabinose	IDA
	Terminal electron receptor	Not reported	IDA
MIGS-6	Habitat	Environment	IDA
MIGS-6.3	Salinity	Tolerates 5 % NaCl	IDA
MIGS-22	Oxygen	Not reported	IDA
MIGS-15	Biotic relationship	Free living	IDA
MIGS-14	Pathogenicity	Non pathogenic, BSL1	NAS
MIGS-4	Geographic location	Daqing, China	IDA
MIGS-5	Sample collection time	March 2012	IDA
MIGS-4.1	Latitude	45°92′N	IDA
MIGS-4.2	Longitude	124°68′E	IDA
MIGS-4.4	Altitude	Not reported	IDA

^a^Evidence codes - *IDA* inferred from direct assay, *TAS* traceable author statement (i.e., a direct report exists in the literature), *NAS* non-traceable author statement (i.e., not directly observed for the living, isolated sample, but based on a generally accepted property for the species, or anecdotal evidence). These evidence codes are the Gene Ontology project [43]

**Fig. 2:**
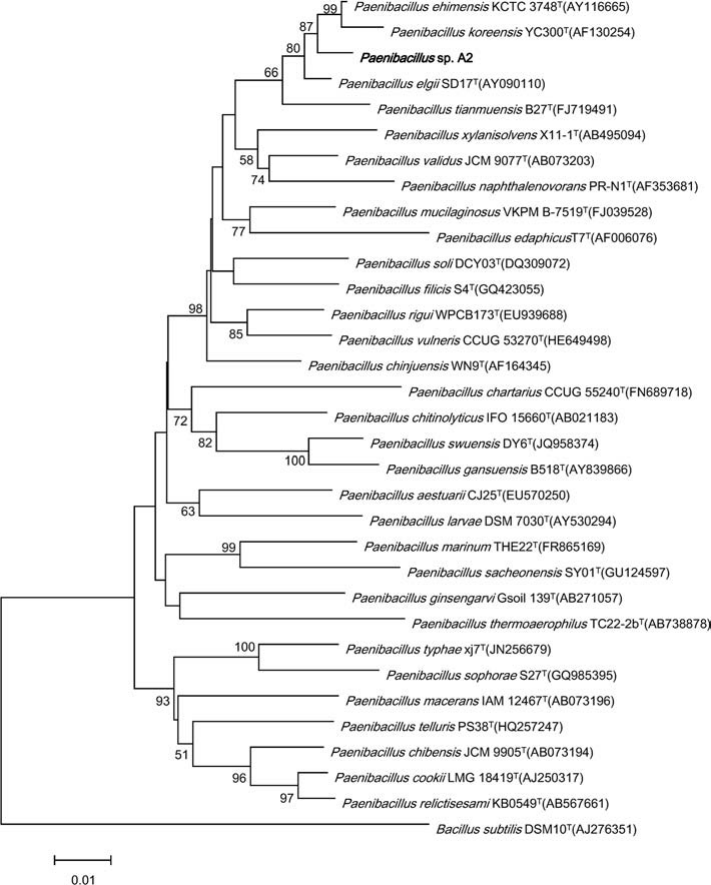
Phylogenetic tree depicting the relationship between *Paenibacillus sp.* strain A2 and other members of the genus *Paenibacillus*. The strains and their corresponding Genbank accession numbers are shown following the organism name and indicated in parentheses. The phylogenetic tree uses 16S rRNA gene sequences aligned by the CLUSTALW [7], and phylogenetic inferences were made using Neighbor-joining method based on Kimura 2-parameter model within the MEGA5 software [8] and rooted with *Bacillus subtilis* strain DSM10^T^ (AJ276351). Bootstrap consensus trees were inferred from 100 replicates, only bootstrap values >50 % were indicated. The scale bar represents 0.01 nucleotide change per nucleotide position

Comparative 16S rRNA gene sequence analysis by BLASTN using the NCBI-NR/NT database revealed 94–99 % sequence similarity to members of genus *Paenibacillus**.* Neighbor-Joining phylogenetic analysis based on Kimura 2-parameter model indicated the *Paenibacillus sp.* strain A2 is most closely related the strain *Paenibacillus ehimensis*KCTC 3748^T^ (AY116665) and *Paenibacillus koreensis* YC300^T^ (AF130254) (Fig. 2).

Biochemical features were tested by using two automated systems, the Vitek2 Compact (bioMérieux, Marcy l’Etoile, France) and Phoenix 100 ID/AST system (Becton Dickinson Company, Sparks, MD. USA). Positive reactions were obtained for glucose, xylose, mannitol and arabinose. Negative reactions were observed for fructose, trehalose, gluconic acid, sucrose, maltose, urea, cellobiose, glucoside, tagatose and maltotriose. This strain was susceptible to gentamicin, ciprofloxacin, levofloxacin, moxifloxacin, tri-methoprim/sulfamethoxazole, amoxicillin, imipenem, meropenem, ciprofloxacin, tigecycline and rifampicin, but resistant to metronidazole.

## Genome sequencing information

### Genome project history

*Paenibacillus sp.* strain A2 was selected for sequencing on the basis of its phylogenetic position and 16S rRNA similarity to other members of the genus *Paenibacillus*, and is part of a microbial diversity study of the oilfield aiming at isolating all bacterial species degrading crude-oil. This whole genome shotgun project of *Paenibacillus sp.* strain A2 is deposited in the Genome On Line Database and the draft genome sequence is deposited at DDBJ/EMBL/GenBank under the accession JFHX00000000 and consists of 180 contigs. A summary of the project information and its association with MIGS version 2.0 compliance are shown in Table 2 [11].

**Table 2:** Project information

MIGS ID	Property	Term
MIGS-31	Finishing quality	High-quality draft
MIGS-28	Libraries used	One pair-end 450 bp library
MIGS-29	Sequencing platforms	Illumina HiSeq 2000
MIGS-31.2	Fold coverage	180.0 × (based on 450 bp library)
MIGS-30	Assemblers	Velvet 1.2.07
MIGS-32	Gene calling method	Glimmer 3.0
	Locus Tag	AA76
	Genbank ID	JFHX00000000
	Genbank Date of Release	April 2, 2014
	GOLD ID	Gi0070607
	BIOPROJECT	PRJNA233560
MIGS-13	Source Material Identifier	CGMCC No. 5647
	Project relevance	Biotechnology

### Growth conditions and genomic DNA preparation

*Paenibacillus sp.* strain A2 was grown aerobically on LB broth, at 37 °C for 16 h. Genomic DNA was extracted using the DNeasy blood and tissue kit (Qiagen, Germany), according to the manufacturer’s recommended protocol. The quantity of DNA was measured by the NanoDrop Spectrophotometer and Cubit. Then 10 μg of DNA was sent to BGI (Shenzhen, China) for sequencing on a Hiseq2000 system.

### Genome sequencing and assembly

One DNA library was generated (450 bp insert size, with the Illumina adapter at both ends detected by Agilent DNA analyzer 2100), then sequenced using an Illumina Hieseq 2000 genomic sequencer, with a 2 × 100 pair end sequencing strategy. Finally, we obtained a total of 5,728,134 M bp and performed the following quality control steps: 1) Reads linked to adapters at both end were considered as sequencing artifacts and removed. 2) Bases with quality index lower than Q20 at both ends were trimmed. 3) Reads with ambiguous bases (N) were removed. 4) Single qualified reads were discarded (In this situation, one read is qualified but its mate is not). Filtered 1378 M clean data were assembled into scaffolds using the Velvet version 1.2.07 with parameters “-scaffolds no” [12], then we use a PAGIT flow [13] to prolong the initial contigs and correct sequencing errors to arrive at a set of improved scaffolds.

### Genome annotation

Predicted genes were identified using Glimmer version 3.0 [14]. tRNAscan-SE version 1.21 [15] was used to find tRNA genes, whereas ribosomal RNAs were found by using RNAmmer version 1.2 [16]. To annotate predicted genes, we used HMMER version 3.0 [17] to align genes against Pfam version 27.0 [18] (only pfam-A was used) to find genes with conserved domains. KAAS server [19] was used to assign translated amino acids into KEGG Orthology [20] with single-directional best hit method. Translated genes were aligned with the COG database [21, 22] using NCBI blastp (hits should have scores no less than 60, e value is no more than 1e-6). To find genes with hypothetical or putative functions, we aligned genes against the NCBI nucleotide sequence database (nt database was downloaded at Sep 20, 2013) by using NCBI blastn, only if hits have identity no less than 0.95, coverage no less than 0.9, and reference genes were annotated as putative or hypothetical. To define genes with a signal peptide, we use SignalP version 4.1 [23] to identify genes using default parameters. TMHMM 2.0 [24] was used to identify genes with transmembrane helices. Prophages and putative phage like elements in the genome were identified using prophage-predicting PHAST [25]. Blast of the three genomes together with strain 2745-2 were performed using blast+program [26]. BLAST Ring Image Generator (BRIG) was used for genome alignment visualization [27].

## Genome properties

**Fig. 3:**
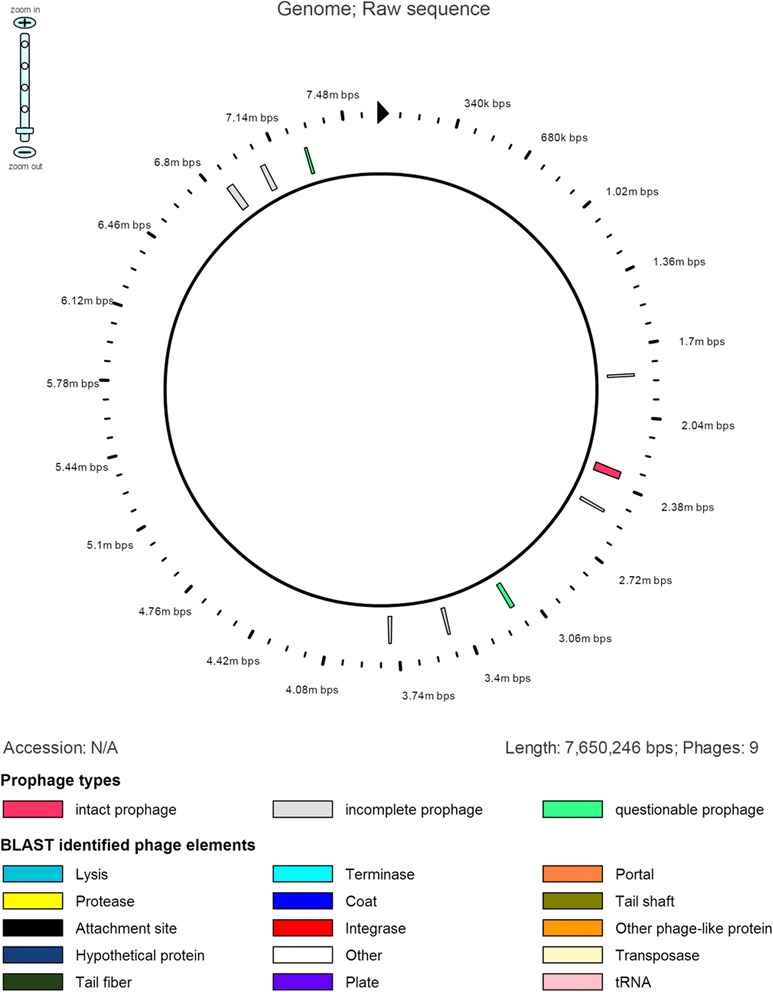
Genomic view of prophage regions identified in the genome of *Paenibacillus sp.* A2

The draft genome sequence of *Paenibacillus sp.* strain A2 revealed a genome size of 7,650,246 bp and a G+C content of 54.2 % (Table 3). The genome contain 7575 coding sequences, 46 tRNAs (excluding 1 pseudo tRNAs) and incomplete rRNA operons (one small subunit rRNA and two large subunit rRNAs). A total of 3112 protein-coding genes were assigned as putative function or hypothetical proteins. Four thousand seven hundred ten genes were categorized into COGs functional groups (including putative or hypothetical genes). The properties and the statistics of the genome are summarized in Tables 3 and 4. Nine prophage regions have been identified in the genome of strain A2 (Fig. 3), including one intact, six incomplete and two questionable regions (Table 5).

**Table 3:** Genome statistics

Attribute	Value	% of Total^a^
Genome size (bp)	7,650,246	100.00
DNA coding region (bp)	6,699,198	87.57
DNA G+C (bp)	4,144,410	54.2
DNA scafflods	180	
Total genes	7578	100.00
Protein coding genes	7575	99.96
RNA genes	49	0.65
Pseudo genes	211	2.78
Genes in internal clusters	203	2.68
Genes with function prediction	5756	76
Genes with Pfam domains	6300	83.16
Genes assigned to COGs	4710	62.15
Genes with signal peptides	405	5.34
Genes with transmembrane helices	1962	25.89
CRISPR repeats	1	–

^a^The total is based on either the size of the genome in base pairs or the total number of protein coding genes in the annotated genome

**Table 4:** Number of genes associated with general COG functional categories

Code	Value	% age	Description
J	209	2.76	Translation, ribosomal structure and biogenesis
A	0	0	RNA processing and modification
K	746	9.85	Transcription
L	195	2.57	Replication, recombination and repair
B	4	0.053	Chromatin structure and dynamics
D	85	1.12	Cell cycle control, mitosis and meiosis
V	258	3.41	Defense mechanisms
T	474	6.26	Signal transduction mechanisms
M	282	3.72	Cell wall/membrane biogenesis
N	128	1.69	Cell motility
Z	2	0.026	Cytoskeleton
U	57	0.75	Intracellular trafficking and secretion
O	191	2.52	Posttranslational modification, protein turnover, chaperones
C	351	4.63	Energy production and conversion
G	787	10.39	Carbohydrate transport and metabolism
E	796	10.51	Amino acid transport and metabolism
F	179	2.36	Nucleotide transport and metabolism
H	265	3.50	Coenzyme transport and metabolism
I	196	2.59	Lipid transport and metabolism
P	549	7.25	Inorganic ion transport and metabolism
Q	261	3.45	Secondary metabolites biosynthesis, transport and catabolism
R	1046	13.81	General function prediction only
S	407	5.37	Function unknown
-	468	6.18	Not in COGs

The total is based on the total number of protein coding genes in the annotated genome

**Table 5:** Summary of prophage regions in *Paenibacillus sp.* A2

Region	Region length	Completeness	CDS	Specific keyword
1	13.2 kb	incomplete	17	tail
2	43.5 kb	intact	55	tail, plate, capsid, protease, portal, terminase, integrase, transposase
3	16.9 kb	incomplete	16	tail
4	23.6 kb	questionable	23	tail, capsid, head, portal, terminase
5	20.2 kb	incomplete	21	terminase, portal, head, capsid, tail
6	19.3 kb	incomplete	21	tail
7	37.7 kb	incomplete	24	integrase, tail
8	30.5 kb	incomplete	25	integrase
9	15.9 kb	questionable	22	tail,lysin, plate

## Insights from the genome sequence

**Fig. 4:**
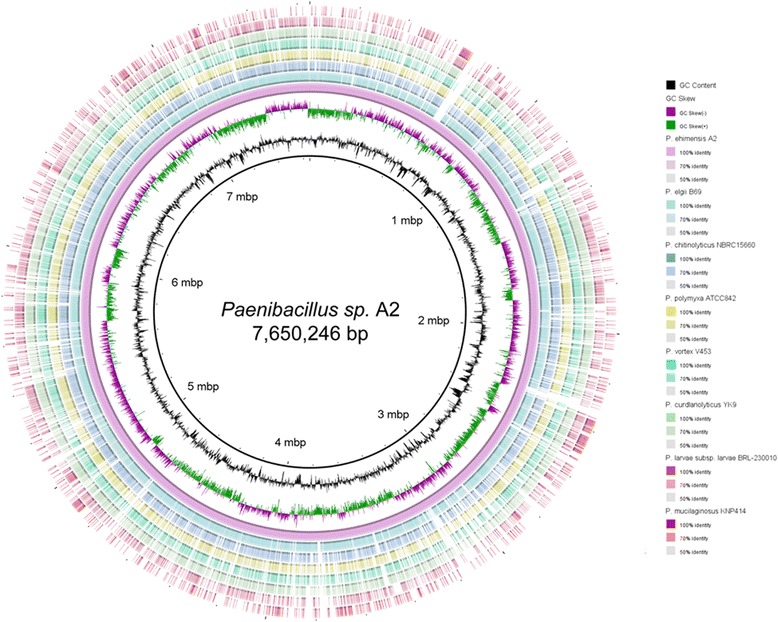
Circular representation of seven draft *Paenibacillus* genomes compared against *Paenibaciluus sp.* A2. The inner rings show GC content (black) and GC skew (purple/green). The remaining rings show BLASTn results of each genome against *P. ehimensis* A2 (JFHX01000001.1) using the BRIG program. The strains used in the BLASTn were *P. elgii* B69 (AFHW01000001.1), *P. chitinolyticus* NBRC15660 (BBJT01000001.1), *P. polymyxa* ATCC842 (AFOX01000001.1), *P. vortex* V453 (ADHJ01000001.1), *P. curdlanolyticus* YK9 (AEDD01000001.1), *P. larvae* subsp. *larvae* BRL-230010 (AARF01000001.1) and *P. mucilaginosus* KNP414 (CP002869.1)

*Paenibacillus sp.* strain A2 grows aerobically with tetradecane and hexadecane as the sole carbon and energy source, and has capability of degrading alkanesulfonate suggesting that it has developed a number of evolutionary strategies that allow for habitat adaptation. To identify pathways associated with niche adaptation to a petroleum reservoir, we explored the genome content for genes associated with hydrocarbon and sulfur metabolism (Table 6). Alkane monooxygenases have been proposed as one of the two unrelated classes of enzymes responsible for the aerobic transformation of midchain-length n-alkanes (C5 to C16) and in some cases even longer alkanes [28]. Sulfate transporters and alkanesulfonate transporter have been shown to play an essential role in metabolizing organosulfur compounds [29, 30]. Based on this knowledge, the genome sequence of strain A2 provides the basis to elucidate its genetic basis for crude oil degradation and adaptation to the petroleum reservoir. BLAST search of nucleotide sequence between strain A2 and other seven *Paenibacillus* species showed that A2 has highest similarity with *Paenibacillus elgii* B69, which is consistent with the 16 s rRNA sequence alignment (Fig. 4).

**Table 6:** Summary of proteins involved in hydrocarbon and sulfur metabolisms

Protein	Start	Stop	Protein product	Length	Description
1	3628875	3629657	WP_025849555.1	260	alkanesulfonate transporter permease subunit
2	3629626	3630852	WP_025849556.1	408	alkanesulfonate monooxygenase
3	6287814	6288827	WP_025846226.1	337	alkane 1-monooxygenase
4	1957755	1958930	WP_025851077.1	391	sulfate adenylyltransferase
5	2493097	2493636	WP_025850577.1	179	adenylylsulfate kinase
6	3634266	3635159	WP_025849561.1	297	sulfate/thiosulfate transporter permease subunit
7	3635181	3636017	WP_025849562.1	278	sulfate transporter
8	3636039	3637142	WP_025849563.1	367	sulfate transporter subunit
9	4328289	4330016	WP_025848942.1	575	sulfate transporter
10	5127629	5128231	WP_025847883.1	200	adenylylsulfate kinase

## Conclusions

Paenibacillus *sp.* strain A2, was isolated from a mixture of formation water and petroleum and has a broad capacity for metabolizing hydrocarbon and organosulfur compounds. To date, no metabolc pathways involved in petroleum degradation or sulfur compounds have been characterized in genus *Paenibacillus*. The genome sequence of the A2 will hopefully provide new insights into the mechanism of degradation and microorganisms adapt to the petroleum reservoir after surfactant flooding. Furthermore, our data takes a step toward a comprehensive genomic catalog of the metabolic diversity of genus *Paenibacillus*.

## Abbreviations

EOR: enhanced oil recovery
PAGIT: post assembly genome improvement toolkit
TMHMM: transmembrane prediction using hidden markov models
